# Sporadic Hirschsprung Disease: Mutational Spectrum and Novel Candidate Genes Revealed by Next-generation Sequencing

**DOI:** 10.1038/s41598-017-14835-6

**Published:** 2017-11-01

**Authors:** Zhen Zhang, Qi Li, Mei Diao, Na Liu, Wei Cheng, Ping Xiao, Jizhen Zou, Lin Su, Kaihui Yu, Jian Wu, Long Li, Qian Jiang

**Affiliations:** 1 Department of General Surgery, Capital Institute of Pediatrics, Beijing, China; 2 MyGenostics Inc, Beijing, China; 3 Department of Surgery, Beijing United Family Hospital, Beijing, China; 4 Department of Paediatrics and Surgery, Faculty of Medicine, Nursing and Health Sciences, Monash University, Victoria, Australia; 5 Department of Pathology, Capital Institute of Pediatrics Affiliated Children’s Hospital, Beijing, China; 6 Reproductive Medicine Center, Clinical College of PLA Affiliated Anhui Medical University, Hefei, China; 7 Department of Pathophysiology, School of Preclinical Sciences, Guangxi Medical University, Nanning, China; 8 Department of Medical Genetics, Beijing Municipal Key Laboratory of Child Development and Nutriomics, Capital Institute of Pediatrics, Beijing, China

## Abstract

Hirschsprung disease (HSCR) is a common cause of functional colonic obstruction in children. The currently available genetic testing is often inadequate as it mainly focuses on *RET* and several other genes, accounting for only 15–20% of cases. To identify novel, potentially pathogenic variants, we isolated a panel of genes from a whole-exome sequencing study and from the published mouse aganglionosis phenotypes, enteric nervous system development, and a literature review. The coding exons of 172 genes were analyzed in 83 sporadic patients using next-generation sequencing. Rare stop-gain, splice-site variants, frameshift and in-frame insertions/deletions and non-synonymous variants (conserved and predicted to be deleterious) were prioritized as the most promising variants to have an effect on HSCR and subjected to burden analysis. GeneMANIA interaction database was used to identify protein–protein interaction-based networks. In addition, 6 genes (*PTPN13*, *PHKB*, *AGL*, *ZFHX3*, *LAMA1*, and *AP3B2*) were prioritized for follow-up studies: both their time-space expression patterns in mouse and human colon showed that they are good candidates for predicting pathogenicity. The results of this study broaden the mutational spectrum of HSCR candidate genes, and they provide an insight into the relative contributions of individual genes to this highly heterogeneous disorder.

## Introduction

Hirschsprung disease (HSCR, MIM# 142623), also known as congenital aganglionosis, is a rare congenital disorder affecting 1 in every 5,000 live births. The pathogenesis is due to the absence of intrinsic ganglion cells in the myenteric and submucosal plexuses of the gastrointestinal tract. Aganglionosis can occur in a short (distal to the sigmoid, accounting for 80% of cases) or long (proximal to the sigmoid, accounting for 15% of the patients) segment of the colon or the entire colon or intestine (5%). Most HSCR cases (70%) are isolated (non-syndromic), with smaller fractions possessing a chromosomal abnormality (12%) or other congenital anomalies (12%)^1^. While highly heritable (>80%), the phenotype varies considerably by gender (4:1 male: female ratio), segment length (short- [S-HSCR], long- [L-HSCR], and total colonic aganglionosis [TCA]), family history, and associated chromosomal abnormalities^2^.

HSCR is characterized by extensive genetic heterogeneity; Up to date, mutations of more than 15 genes have been known to be associated with the disease. With the exception of the *RET* proto-oncogene that is responsible for approximately 50% of familial and up to 15% of sporadic cases, other HSCR genes only individually account for a small percentage of the cases. Genetic screening with traditional approaches, such as direct sequencing, is therefore difficult. A high-throughput and cost-effective method to detect genetic defects is needed. Although whole-exome sequencing has proven to be a powerful tool for discovering novel disease-related genes and mutations in large genomic regions^3–5^, the extensive information, the subsequent arduous data processing, and high cost dramatically limit its wide application, especially in China.

Therefore, in this study, we performed targeted enrichment and next-generation sequencing of 172 candidate genes in a cohort of 83 patients, in order to establish a strategy feasible for the genetic diagnosis of HSCR and explore the mutation spectrum, phenotype-related gene set, and cumulative genetic risk in this population.

## Methods

### Ethical statement

All procedures performed in studies involving human participants were in accordance with the ethical standards of the institutional and/or national research committee and with the 1964 Helsinki declaration and its later amendments or comparable ethical standards. The protocols of this study were reviewed and approved by the Ethics Committee of the Capital Institute of Pediatrics, Beijing, China (Proposal Number SHERLL 2013039) and informed consents were obtained from all participants. For those aged under 18 years of age, written consent was given by their guardians.

### Patients and colon tissue

A total of 83 unrelated, sporadic patients enriched for the most severe form (TCA and the long-segment HSCR) were recruited in our study to maximize the probability of identifying the relevant potential pathogenic variants (genes). All our patients were recruited from the Department of General Surgery, Capital Institute of Pediatrics, Beijing, China between June 2013 and June 2015. All patients were seen by a clinical geneticist to rule out obvious syndromic disorders. Physical examination confirmed the absence of other congenital anomalies. All the probands were diagnosed by barium enema and anorectal manometry before surgical procedures. After surgery, a definite diagnosis was made by pathological examination.

Control colon tissues (neither ischemic nor necrotic) were obtained from two individuals (both males, aged 5 and 14 months) who underwent surgery because of intestinal obstruction and a strangulated inguinal hernia. These patients were confirmed to have no HSCR or other congenital malformation. In addition, samples were collected from 16 HSCR patients (8 males and 8 females; 4/7/5 S-HSCR, L-HSCR, and TCA, average age, 6.9 months) who had been shown to harbor likely gene-disrupting (LGD) mutations in any of the following genes: *PTPN13*, *PHKB*, *AGL*, *ZFHX3*, *LAMA1*, *AP3B2*, or *RET*.

To establish the temporal expression patterns of 6 novel candidate genes, we collected colon specimens from murine fetus (on embryonic (E) days E8.5, E10, E12, and E16) and postnatal (1 and 6 weeks) outbred CD-1^®^(ICR) IGS mice (strain code: 201). The day on which a vaginal plug was evident was taken to be E0.5. All animal studies were performed following the protocols approved by the Animal Care and Use Committee of the Capital Institute of Pediatrics. Three mice were used for each time-point and samples were mixed and gene expression was assessed using real-time PCR (primers available on request). All tissues were frozen and stored at −80 °C immediately after surgery or dissection. Total RNA was extracted from the colonic tissues using PureLink RNA Mini Kit (Thermo Fisher Scientific, Waltham, MA) according to the manufacturer’s protocol. cDNA syntheses involved 1 μg RNA with the SuperScript™III First-Strand Synthesis SuperMix for qRT-PCR (Invitrogen, Carlsbad, CA). *Gapdh* was used as the loading control. The reaction program was: pre-denaturation at 50 °C 2 min and 95 °C 10 min, followed by 40 cycles of 15 s of denaturation at 95 °C, 60 s of annealing at 60 °C. The amplification process was followed by a melting curve analysis and the threshold cycle (Ct) value was recorded. The specificity of each real-time PCR product was evaluated with a dissociation curve. The relative mRNA levels for each sample were calculated using the 2^−△Ct^ method.

### Target gene selection

Altogether 172 candidate genes were selected for the current study based on the following evidence after removing redundancy: (1) human linkage analysis or human association studies showing that they play a role in HSCR (n = 15); (2) large recurrent copy number variations in humans or having significantly altered gene expression in comparisons of the gastrointestinal tract of *Ret* ^+/+^ (wild-type) *versus Ret* ^−/−^ (null) mice (n = 11); (3) those recognized in a mouse aganglionosis phenotype (n = 9); (4) those with >5 single nucleotide variants (SNVs) identified among 211 unrelated European-American HSCR probands in a whole-exome sequencing project (manuscript in preparation) and confirmed to be expressed in the enteric nervous system (ENS) based on the RNAseq data from the Illumina Human Body Map 2.0 project (n = 108); (5) new HSCR candidate genes with significantly more deleterious mutations in phenotype-enriched individuals in the whole-exome sequencing project although not expressed in the ENS (n = 13); and (6) those suggested to play important roles in the development of the ENS (n = 18). Detailed information on these HSCR candidate genes and their chromosome locations, Entrez gene IDs, protein sizes (numbers of amino-acids), as well as the selection criteria are provided in Supp. Table S1.

### Library construction, enrichment, and targeted next-generation sequencing

Genomic DNA was extracted from patients’ whole blood using a DNA extraction kit (DNeasy Blood & Tissue Kit, Qiagen, Shanghai, China) according to the manufacturer’s instructions. DNA quantification was performed using NanoDrop 1000 (Thermo Fisher Scientific, Wilmington, DE). A minimum of 3 μg DNA was used for construction of the indexed Illumina libraries following the manufacturer’s protocol. A final library size of 350–400 bp, including adapter sequences, was selected.

Candidate disease genes (n = 172) were enriched by a gene capture strategy using a GenCap custom enrichment kit (MyGenostics, Beijing, China) according to previously-described methods^6,7^. Briefly, 1 μg of DNA library was mixed with BL buffer and a GenCap hypercholesterolemia probe (MyGenostics, Beijing, China) and heated in a polymerase chain reaction (PCR) cycler at 95 °C for 7 min and 65 °C for 2 min. HY buffer (23 μL pre-warmed to 65 °C; MyGenostics) was then added and the mixture was held at 65 °C for 22 h for hybridization. MyOne beads (50 μL; Life Technology, Carlsbad, CA) were washed thrice in 500 μL binding buffer (1x) and re-suspended in 80 μL binding buffer (1x); then 64 μL binding buffer (2x) was added, the mixture transferred into a tube containing 80 μL MyOne beads, and spun for 1 h on a rotator. The beads were then washed once with WB1 buffer at room temperature for 15 min and thrice with WB3 buffer at 65 °C for 15 min. Elution buffer was used to elute the bound DNA, which was amplified as follows: 98 °C for 30 s; 98 °C for 25 s, 65 °C for 30 s, and 72 °C for 30 s (15 cycles); 72 °C for 5 min. We purified the PCR product using SPRI beads (Beckman Coulter, Indianapolis, IN) following the manufacturer’s protocol. Enrichment libraries were sequenced on an Illumina HiSeq. 2000 sequencer (Illumina, San Diego, CA) for 100-bp paired reads.

### Variant filtering and classification

After sequencing, we retrieved high-quality reads from raw reads by filtering out low-quality reads (mapping qualities <30, total mapping quality zero reads <4, long homo-polymer run >5, approximate read depth <5, QUAL <50.0, phred-scaled p-value using Fisher’s exact test to detect strand bias >10.0), and adaptor sequences using the Solexa QA package^8^ and the cutadapt program (http://code.google.com/p/cutadapt/, v1.9.1). We used the SOAPaligner program^9^ to align the clean read sequences to the human reference genome (UCSC Genome Browser hg19). After removing duplicates with Picard software (v1.119)^10^, single-nucleotide polymorphisms (SNPs) were identified using the SOAPsnp program (http://soap.genomics.org.cn/soapsnp.html)^9^. Subsequently, reads were realigned to the reference genome using the Burrows–Wheeler alignment program (0.7.12-r1044)^11^, and insertions or deletions (InDels) were detected by HaplotypeCaller of GATK software (https://software.broadinstitute.org/gatk/, GATK-3.5) and filtered by VariantFiltration of GATK software^12^. We annotated the identified SNPs and InDels using the exome-assistant program. Short read alignment and candidate SNP and InDel validation were performed using MagicViewer^13^. We used the PolyPhen, SIFT, and MutationTaster algorithms to evaluate non-synonymous variants to determine pathogenicity.

### Mutation validation and inheritance ascertainment

We performed Sanger sequencing for all the identified LGD_strict_ (likely gene-disrupting) mutations (including stop-gain, frameshift, and canonical splice-site variants) among the probands. DNA from parents and other family members was also examined to ascertain the inheritance pattern and segregation of the variant where applicable. Sanger sequencing was performed on genomic DNA from peripheral blood. Primers for Sanger sequencing (sequences on request) were designed with Primer 3 software and sequenced on an ABI 3730 sequencer (Applied Biosystems, Life Technologies). The data were analyzed with Sequencher 5.4.1 (Gene Codes Corp., Ann Arbor, MI).

### Bioinformatics analysis

To evaluate the roles of the candidate genes, their annotations were studied using the Database for Annotation, Visualization and Integrated Discovery (DAVID) *via* three GO (Gene Ontology) classifications: cellular components, biological processes, and molecular functions^14^. A list of 13 LGD_strict_ variant-associated genes was analyzed. The significant functional categories were selected in the functional annotation cluster analysis. The enrichment was quantified using Fisher’s exact test. Bonferroni correction was used to adjust for multiple testing. Gene burden tests for the filtered variants in the LGD_strict_ and LGD_broad_ groups were performed separately by analyzing 83 HSCR patients and 208 control samples collected from the Chinese population in the 1000 genome project. Gene-based association tests were performed using only rare variants with a minor allele frequency <1% with the sequence kernel association test^15^ implemented in the sequence kernel association test (SKAT version 1.1.2) package in R. The false discovery rate (FDR) values were determined using the Bonferroni-corrected *P* values for multiple tests. Cytoscape with the GeneMANIA plugin^16^ was used to identify the genes most related to the LGD_strict_ gene set. The GeneMANIA core dataset of co-expression, co-localization, pathway, genetic, physical, and predicted interactions was subjected to protein-protein interaction network analysis using default parameters.

### Data availability statement

The datasets generated during and/or analysed during the current study are available from the corresponding author on reasonable request.

## Results

### Patient characteristics

The clinical characteristics of the 83 HSCR patients (male/female: 51/32) are outlined in Table 1. All patients were classified based on the length of aganglionosis into three categories: S-HSCR, L-HSCR, and TCA (32/19/32). In addition, the majority of the children (60/83, 73%) underwent surgery before 12 months of age.

**Table 1:** Clinical categorization of 83 HSCR patients.

	*Segment length*			*Sub-total*
	*Short (S-HSCR)*	*Long (L-HSCR)*	*Total colonic aganglionosis (TCA)*	
Gender				
Male	12	14	25	51
Female	20	5	7	32
Age at surgery (months)				
0–6	16	8	18	42
6–12	4	5	9	18
12–36	7	3	4	14
>36	5	3	1	9

### Variant identification and classification

**Figure 1:**
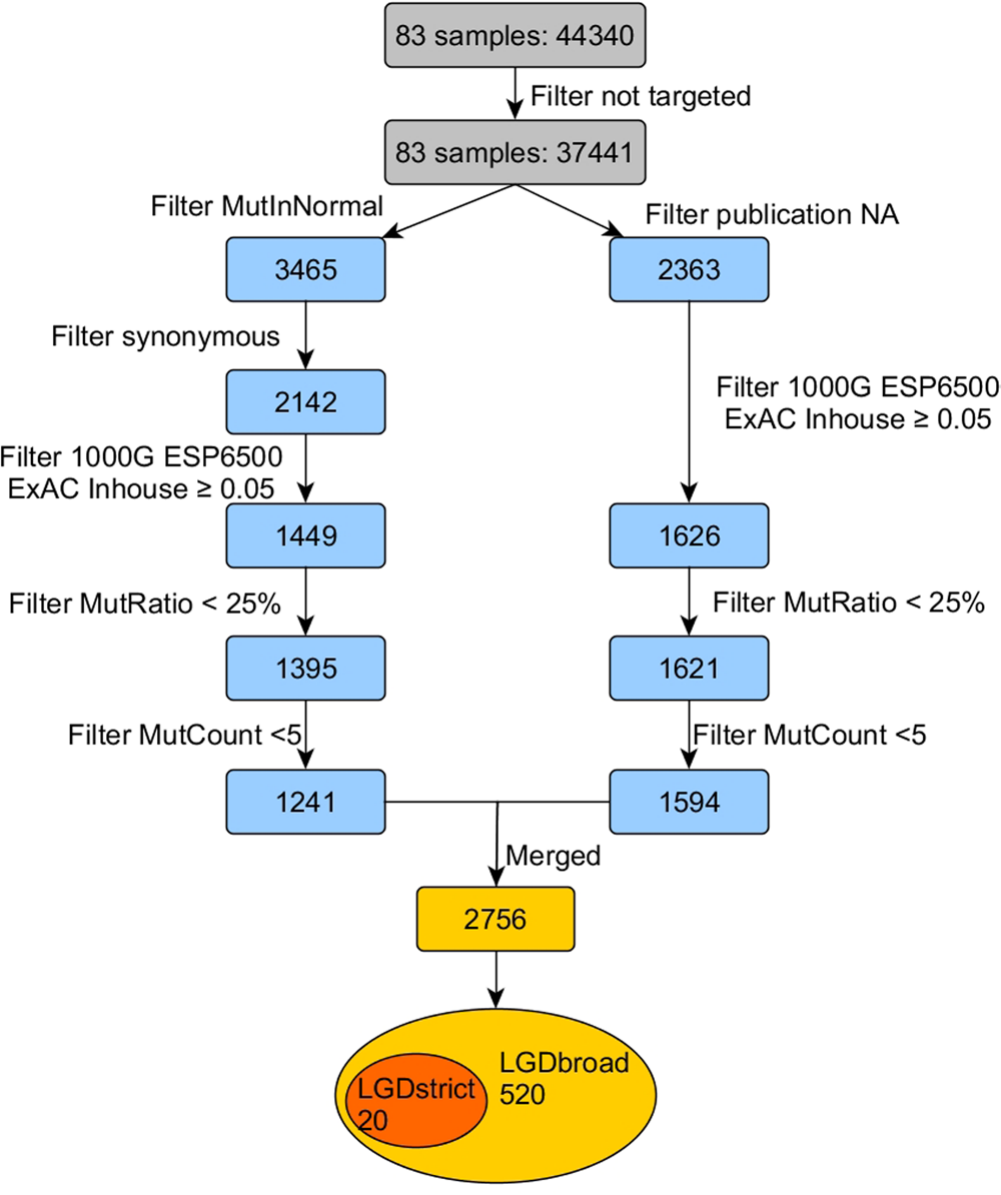
Schematic of the analytical workflow. A two-step variant filtering and prioritization approach was used to select candidate variants for disease-causing mutations. In the first step (on the left), all variants present in the in-house control cohort (MutInNormal) and all synonymous variants were excluded; while in the alternative step (on the right), all variants that had ever been reported were retained, regardless of their effects. Later, variants left from either step were further filtered against high frequency (variants with an alternative allele frequency ≥0.05 in either the 1000 Genome Project, the NHLBI Exome Sequencing Project, the Exome Aggregation Consortium data and an internal exome database of ~500 individuals) and low quality or potential false positives (MutRatio <25% and MutCount <5), before being merged into a non-redundant, integrated list of 2756. Two categories of variants were further extracted with the following definitions: LGD_strict_ refers to stop-gain, canonical splice-site (±2) variants and frameshift InDels. LGD_broad_ refers to stop-gain, splice region (±5) variants, frameshift InDels, in-frame InDels, and non-synonymous (missense) variants predicted to be damaging by at least three bioinformatics tools.

On average, 276.2 million reads of 100-bp length were generated per patient sample (except for HSCR0042, a male patient with TCA). A minimum 20-fold coverage per base was achieved on average for 85.2% of the target region at a mean coverage of 266 reads (Basic QC metrics are shown in Supp. Table S2). Regions with insufficient coverage were mostly recurrent and usually characterized by a high GC content. In total, 37,441 variants (Supp. Fig. S1A) were identified among the 83 patients after filtering the off-target changes. A two-step variant filtering and prioritization approach (Fig. 1) was applied to select candidate variants for disease-causing mutations. For the first step, all variants present in the in-house control cohorts (MutInNormal, number of ~500) and all synonymous variants were excluded. Alternatively, all variants that had been reported in HGMD version October 2016 (and thus may be plausible candidates for human disease) were retained, regardless of their effects. Then, variants left from either step were further filtered against high frequency (variants with an alternative allele frequency ≥0.05 in either the 1000 Genome Project, the NHLBI Exome Sequencing Project (ESP6500), the Exome Aggregation Consortium data (http://exac.broadinstitute.org/) or an internal exome database of ~500 individuals) and low quality or potential false positives (MutRatio <25% and MutCount <5, MutRatio = MutCount/coverage; MutCount is the total number of reads indicating certain kind of alternative allele), before being merged into a non-redundant, integrated list (Supp. Fig. S1B). Two categories of variants were further extracted from these 2756 variants with the following definition: LGD_strict_ includes stop-gain, canonical splice-site (±2) variants, and frameshift InDels. LGD_broad_ refers to stop-gain, splice region (±5) variants, frameshift InDels, in-frame InDels, and non-synonymous (missense) variants predicted to be damaging by at least three bioinformatics tools out of five (SIFT, PolyPhen, MetalR, CADD and M-Cap).

We found 19 LGD_strict_ variants in 13 genes (*RET*, *TLX2*, *PTPN13*, *SACS*, *PLEKHH1*, *TRAP1*, *EXO1*, *ZEB2*, *AGL*, *SEMA3D*, *PHKB*, *HHIPL2*, and *ENO3*) among the 83 patients (Table 2); these consist of 5 frameshift, 13 stop-gain, and 1 canonical splice-site variant. One stop-gain mutation (p. E560X) in *FARP1* (Supp. Fig. S2) was not validated by Sanger sequencing and was eliminated from the original list of 20. Notably, *RET* was the only recurrent disrupting gene affecting 7 unrelated isolated HSCR patients. This discovery rate (8.4%) was within expectation but the inheritance pattern was not anticipated: all 5 for which parental DNA was available (p.E252X, p.Y263X, p.R770X, p.Q860X, and c.2802-2 A > G) were *de novo* mutations. The *de novo* rate in the major disease gene *RET* was at least 71.4% (5/7), and could be as high as 100% (7/7) because blood sample from the parents of two probands was not available. In addition to *RET*, truncating mutations were detected in two other known HSCR genes: a *de novo* stop-gain mutation (p.R278X) in *ZEB2* and a paternally-inherited nonsense mutation (p.R760X) in *SEMA3D*. The c.832 C > T substitution creates a premature stop codon (CGA > TGA) in exon 7 of the *ZEB2* gene (NM_014795.3) and its presence is consistent with a diagnosis of Mowat-Wilson syndrome. Retrospective medical review revealed a mild facial gestalt and a congenital heart defect in our female patient supporting this diagnosis. Ten novel mutations were discovered in 10 new candidate genes, including 4 frameshift and 6 stop-gain variants: 5/6 of the stop-gain mutations were predicted to be disease-causing by MutationTaster with an average GERP (genomic evolutionary rate profiling) score of 3.95. Interestingly, all the novel mutations for which an inheritance pattern was ascertainable were inherited from asymptomatic mothers. Most of the variants (13/19) were absent from publically available databases (dbSNP, 1000 Genome Project, the NHLBI Exome Sequencing Project and ExAC) and our in-house control. In order to overcome the shortcoming of unidirectional analysis on only cases, we also incorporated data from 316 exome-sequenced Chinese controls (acknowledgements to the MyGenostics Inc.) and processed the variants for each of the 172 genes with the same two-step variant filtering and prioritization approach as for cases. In the Supp. Table S3, we provided the comparison of the frequency, type and novelty of all likely gene-disrupting variants detected in these 13 genes in both HSCR cases (number of 83) and controls (number of 316).

**Table 2:** List of 19 LGD_strict_ variants identified among 83 Chinese HSCR patients and validated by Sanger sequencing.

*Patient ID*	*Gene*	*Chromosome position*	rs ID	*Nucleotide change* ^*a*^	*Amino-acid change*	*Mutation Taster* ^*b*^	*GERP* ^*c*^	*Frequency*				*Gender*	*Type* ^*e*^	*Inheritance*
								1000G	ESP6500	ExAC^d^	Inhouse			
HSCR0058	*TLX2*	Chr2: 74742218	—	c.293insG	p.A98Gfs*268	—	—	0	0	0	0	Male	S	—
HSCR0116	*RET*	Chr10: 43600528	—	c.754G > T	p.E252X	DC	2.1	0	0	0	0	Male	TCA	*de novo*
HSCR0117	*RET*	Chr10: 43615164	—	c.2578C > T	p.Q860X	DC	4.42	0	0	0	0	Male	TCA	*de novo*
HSCR0129	*PTPN13*	Chr4: 87728936	—	c.6396G > A	p.W2132X	DC	5.68	0	0	0	0	Female	L	Mother
HSCR0129	*RET*	Chr10: 43600563	—	c.789C > G	p.Y263X	DC	−3.43	0	0	0	0	Female	L	*de novo*
HSCR0013	*RET*	Chr10: 43613869	—	c.2333delT	p.V778Afs*1	—	—	0	0	0	0	Male	S	—
HSCR0009	*SACS*	Chr13: 23912864	rs761184491	c.4710delA	p.K1570Nfs*7	—	—	0	0	0.0003	0	Female	S	—
HSCR0022	*PLEKHH1*	Chr14: 68053899	rs111462449	c.4042C > T	p.R1348X	PO	3.11	0.01	0.008626	0.0076	0	Male	L	—
HSCR0033	*TRAP1*	Chr16: 3740957	—	c.118delA	p.T40Qfs*76	—	—	0	0	0	0	Male	TCA	Mother
HSCR0034	*EXO1*	Chr1: 242042155	—	c.1619C > G	p.S540X	DC	3.26	0	0	0	0	Female	L	—
HSCR0038	*ZEB2*	Chr2: 145158778	rs587784571	c.832C > T	p.R278X	DC	4.43	0	0	0	0	Female	L	*de novo*
HSCR0055	*AGL*	Chr1: 100327076	rs781580050	c.49C > T	p.R17X	DC	2.92	0	0	0.00000831	0	Female	S	Mother
HSCR0055	*RET*	Chr10: 43619117	—	c.2802-2A > G	-	DC	5.33	0	0	0	0	Female	S	*de novo*
HSCR0057	*SEMA3D*	Chr7: 84628812	—	c.2278C > T	p.R760X	DC	5.05	0	0	0	0	Male	L	Father
HSCR0070	*PHKB*	Chr16: 47694459	—	c.2014C > T	p.R672X	DC	3.39	0	0	0	0	Female	S	—
HSCR0074	*RET*	Chr10: 43613844	rs775711017	c.2308C > T	p.R770X	DC	3.49	0	0	0.000008277	0	Male	TCA	*de novo*
HSCR0082	*HHIPL2*	Chr1: 222721107	rs748262144	c.279_280insGA	p.H94Dfs*58	—	—	0	0	0.00002486	0.001997	Male	TCA	—
HSCR0146	*RET*	Chr10: 43596087	—	c.254G > A	p.W85X	DC	5.51	0	0	0	0	Male	TCA	—
HSCR0146	*ENO3*	Chr17: 4859895	rs550460218	c.1095G > A	p.W365X	DC	5.32	0.000199681	0	0.0001	0	Male	TCA	—

^a^Nucleotide numbering of the exonic variants reflects cDNA numbering with + 1 corresponding to the A of the ATG translation initiation codon in the reference sequence, specifically, RefSeq NM_016170 for *TLX2*, RefSeq NM_020630 for *RET*, RefSeq NM_080684 for *PTPN13*, RefSeq NM_001278055 for *SACS*, RefSeq NM_020715 for *PLEKHH1*, RefSeq NM_016292 for *TRAP1*, RefSeq NM_003686 for *EXO1*, RefSeq NM_001171653 for *ZEB2*, RefSeq NM_000645 for *AGL*, RefSeq NM_152754 for *SEMA3D*, RefSeq NM_000293 for *PHKB*, RefSeq NM_024746 for *HHIPL2* and RefSeq NM_001976 for *ENO3*.

^b^DC, disease-causing; PO, polymorphism.

^c^GERP, Genomic Evolutionary Rate Profiling. Score ranges from −15 (not conserved) to 7 (conserved).

^d^The allele frequency is based on the Exome Aggregation Consortium data (http://exac.broadinstitute.org/) of all individuals.

^e^S, short-segment HSCR; L, long-segment HSCR; TCA, total colonic aganglionosis.

**Figure 2:**
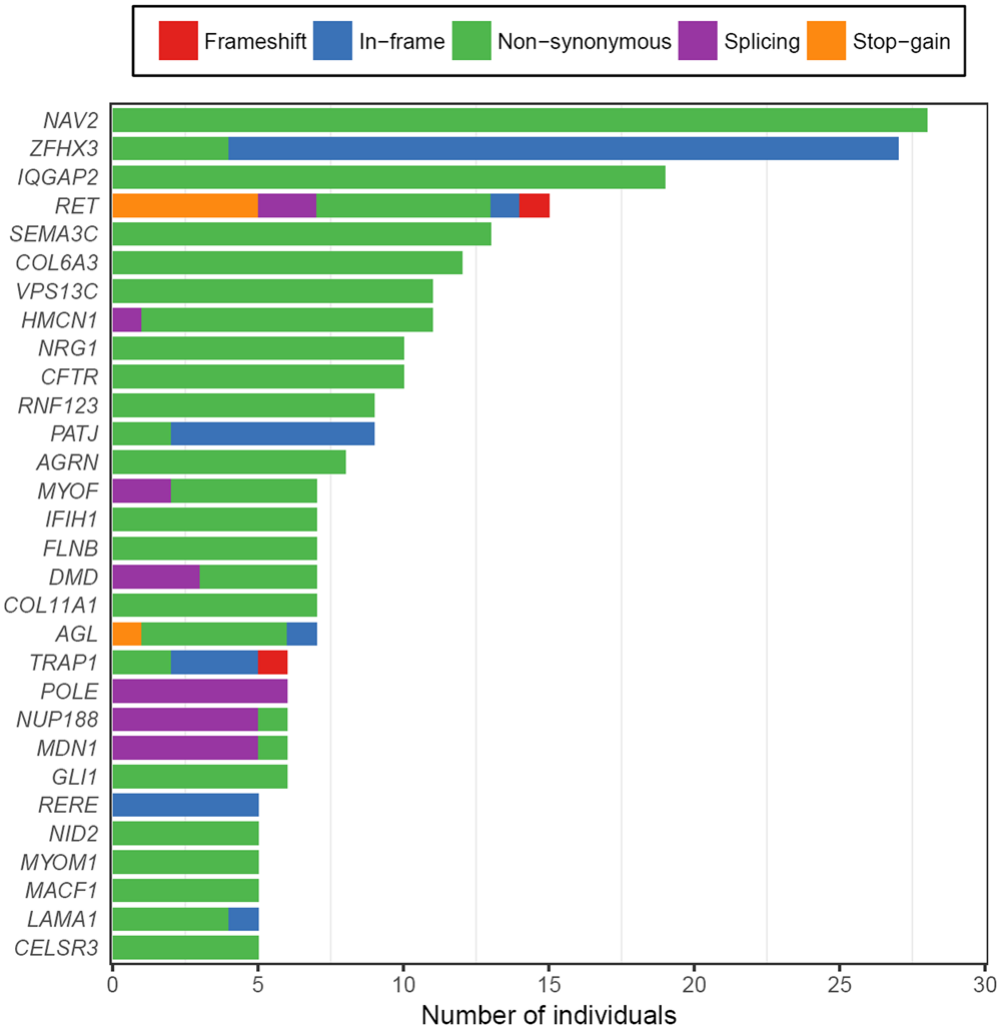
Top 30 HSCR-implicated genes in which LGD_broad_ variants were detected in several patients. The numbers of HSCR patients who carried a candidate variant per gene are shown on the x axis. LGD_broad_ candidates consist of five categories of rare variants: frameshift indels (red), inframe indels (blue), splice region ( ± ) variants (purple), stop-gain variants (orange), and non-synonymous variants (green) predicted to be damaging by at least three bioinformatics tools.

To further our genetic understanding of the disease, we also collected candidate variants following a less stringent criterion: a total of 520 LGD_broad_ variants were discovered and their distributions across genes and patients were investigated. All 83 patients were revealed to harbor LGD_broad_ variants, with a minimum of 1 and a maximum of 12 in a single patient (median = 6, Supp. Fig. S3). The top 30 HSCR-implicated genes are shown in Fig. 2. Ten genes had variants in >10 patients: *NAV2*, *ZFHX3*, *IQGAP2*, *RET*, *SEMA3C*, *COL6A3*, *VPS13C*, *HMCN1*, *NRG1* and *CFTR*. Of these, both novel genes that affected more HSCR patients than *RET* are possibly implicated in the phenotype: (1) *ZFHX3* encodes a transcription factor with multiple homeodomains and zinc finger motifs and regulates myogenic and neuronal differentiation; and (2) *IQGAP2* encodes a member of the IQGAP (IQ motif-containing GTPase-activating protein) family that interacts with components of the cytoskeleton, cell adhesion molecules, and several signaling molecules to regulate cell morphology and motility.

### LGD variants analysis

**Figure 3:**
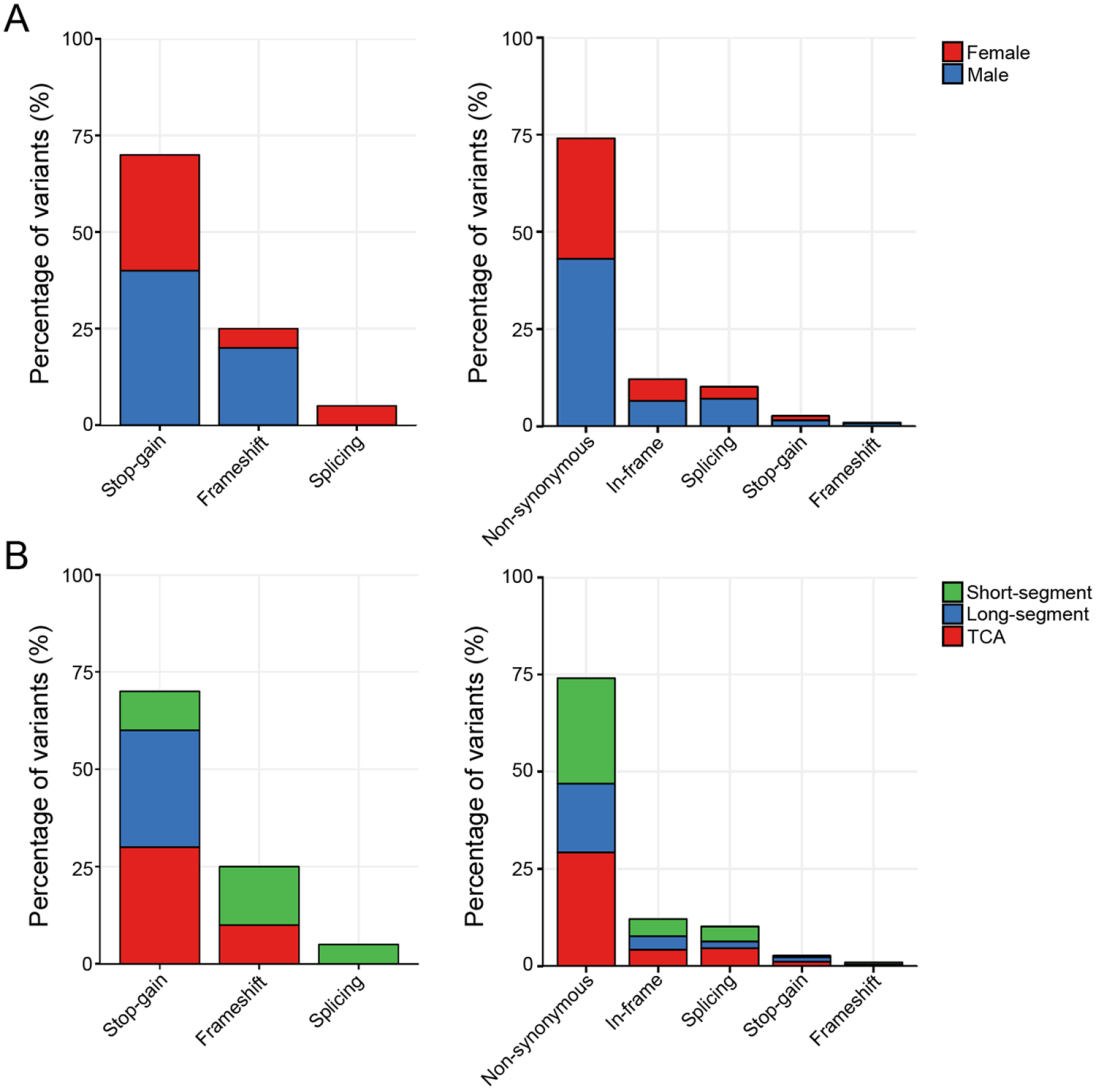
Distribution of the LGD variants across gender and segment length. LGD_strict_ variants occurred slightly more frequently in males than in females (57.9% *vs* 42.1%). (**A**) similar proportion was found for the LGD_broad_ variants (59.0% *vs* 41.0%, A). In contrast, the most severe phenotype group, namely L-HSCR and TCA, dominated both the LGD_strict_ and LGD_broad_ variants: 13 LGD_strict_ variants (68.4%) and 330 LGD_broad_ variants (63.6%) were present in the L-HSCR + TCA group (**B**). S, L, and TCA denote short-segment HSCR, long-segment HSCR, and total colonic aganglionosis.

LGD_strict_ variants occurred more frequently in males than females (57.9% *vs* 42.1%), and a similar proportion was found for the LGD_broad_ variants (59.0% *vs* 41.0%, Fig. 3A). In addition, the most severe phenotype groups, L-HSCR and TCA, dominated both the LGD_strict_ and LGD_broad_ variants: 13 LGD_strict_ variants (68.4%) and 330 LGD_broad_ variants (63.6%) were present in the L-HSCR + TCA group (Fig. 3B). These variant compositions are not unexpected since both the male patients and the L-HSCR + TCA group patients are significantly more than their counterparts among the 83 subjects.

For primary analysis of the two sets of candidate variants, we first chose a popular approach, the sequence kernel association test, to assess the collective effect of genetic coding/splicing mutations in each individual gene to pinpoint good candidates contributing to HSCR. Among the 13 genes in which LGD_strict_ variants were detected, only *RET* displayed nominally significant enrichment for rare damaging variants and passed the burden test (*P* = 3.35E-05, FDR = 0.00047, other data not shown). However, when we performed the gene-based analyses on all LGD_broad_ variants of disrupting genes, many reached statistical significance (Supp. Table S4). Specifically, up to 67 genes with multiple SNVs among HSCR samples were significantly associated with the phenotype, with an FDR ≤0.05. Further functional annotation of the list of genes revealed strong relationships with the phenotype, such as *LAMA1*, *IFIH1*, and *AP3B2*, and these may be new HSCR candidate genes.

**Figure 4:**
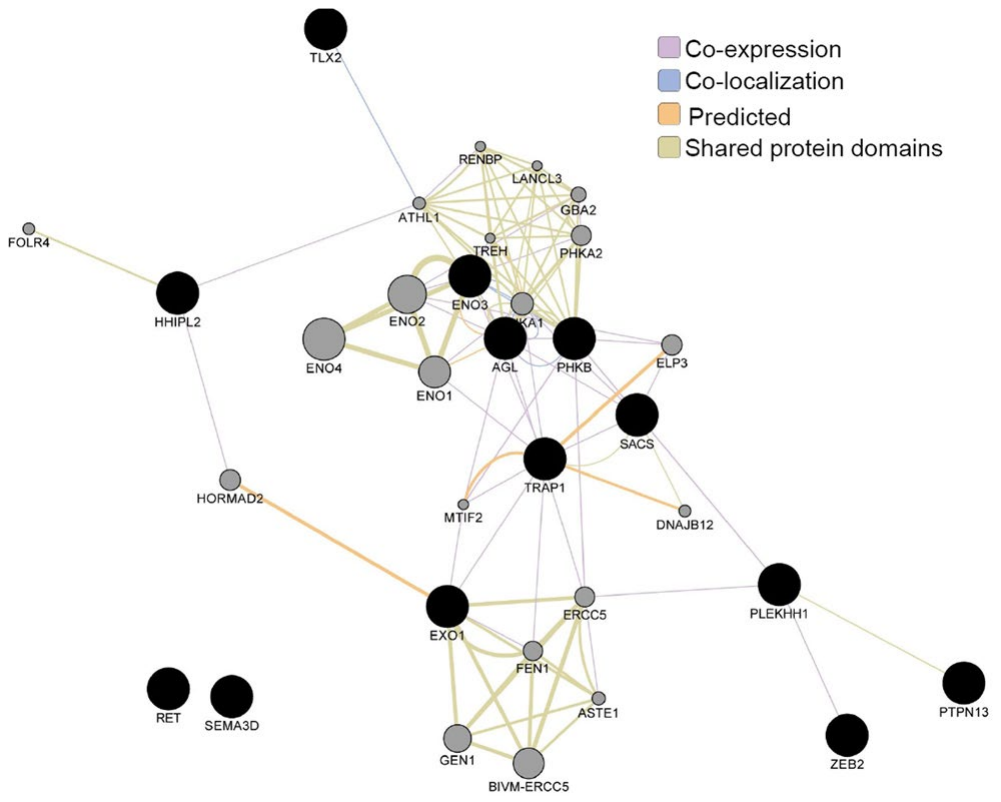
Protein–protein interaction network of the LGD_strict_ variant-associated gene set identified using GeneMANIA. We used GeneMANIA to identify the genes most related to the LGD_strict_ gene set and to search for a possible protein–protein interaction network among them. The query genes (black circles) were assigned a label value of 1. Label propagation was then applied to the entire network and the resulting labels were saved as the score attributed in the node table. This score indicated the relevance of each gene to the original list based on the selected networks. Higher scores (larger circles) indicate genes that are more likely to be functionally related. This analysis found an interconnected network of 11 out of 13 proteins through either co-expression, co-localization, shared protein domains, or predicted interactions.

Given the limited power of our sample size to detect effects at the variant or gene level, we next shifted our focus to the analysis of gene sets and gene networks, which could aggregate a sufficient number of rare variants for an enrichment analysis to reach statistical significance. First, to gain insights into the functions of the identified genes, we tested the probability of the 13 LGD_strict_ variant-associated genes clustering into a specific GO term or a particular pathway. The most important results of the functional annotation clustering analysis are listed in Table 3. The HSCR genes tended to enrich in the regulation of ENS development, glucose, hexose, monosaccharide metabolism, tissue morphogenesis, neural crest cell differentiation, and development. Unfortunately, all gene sets showed only nominal evidence of association and none of them remained significant after accounting for multiple testing of the different frequency and annotation categories *via* permutation. Next, we used GeneMANIA to identify the genes most related to the LGD_strict_ gene set and to search for a possible protein-protein interaction network among them. This analysis revealed an interconnected network of 11 out of 13 proteins through either co-expression, co-localization, shared protein domains, or predicted interactions (Fig. 4).

**Table 3:** Significant findings of functional annotation cluster analysis for LGD_strict_ genes with a nominal *P* < 0.05.

*Category*	*Term*	*Count*	%	*Genes*	*Fold enrichment*	*P*	*Bonferroni*
GOTERM_BP_FAT	GO:0048484~enteric nervous system development	2	15.4	*RET, TLX2*	450.93	3.99E-03	0.564
GOTERM_BP_FAT	GO:0006006~glucose metabolic process	3	23.1	*PHKB, ENO3, AGL*	26.53	4.34E-03	0.596
GOTERM_BP_FAT	GO:0048729~tissue morphogenesis	3	23.1	*RET, ZEB2, TLX2*	22.55	5.96E-03	0.712
GOTERM_BP_FAT	GO:0019318~hexose metabolic process	3	23.1	*PHKB, ENO3, AGL*	21.14	6.76E-03	0.756
GOTERM_BP_FAT	GO:0005996~monosaccharide metabolic process	3	23.1	*PHKB, ENO3, AGL*	18.28	8.95E-03	0.846
GOTERM_BP_FAT	GO:0048483~autonomic nervous system development	2	15.4	*RET, TLX2*	142.40	1.26E-02	0.928
GOTERM_BP_FAT	GO:0001755~neural crest cell migration	2	15.4	*RET, ZEB2*	117.63	1.52E-02	0.959
GOTERM_BP_FAT	GO:0048598~embryonic morphogenesis	3	23.1	*RET, ZEB2, TLX2*	13.22	1.66E-02	0.969
GOTERM_BP_FAT	GO:0006091~generation of precursor metabolites and energy	3	23.1	*PHKB, ENO3, AGL*	12.97	1.73E-02	0.973
GOTERM_BP_FAT	GO:0014033~neural crest cell differentiation	2	15.4	*RET, ZEB2*	81.99	2.17E-02	0.990
GOTERM_BP_FAT	GO:0014032~neural crest cell development	2	15.4	*RET, ZEB2*	81.99	2.17E-02	0.990
GOTERM_BP_FAT	GO:0005977~glycogen metabolic process	2	15.4	*PHKB, AGL*	77.30	2.31E-02	0.992
GOTERM_BP_FAT	GO:0006073~cellular glucan metabolic process	2	15.4	*PHKB, AGL*	75.16	2.37E-02	0.993
GOTERM_BP_FAT	GO:0044042~glucan metabolic process	2	15.4	*PHKB, AGL*	75.16	2.37E-02	0.993
GOTERM_BP_FAT	GO:0001667~ameboidal cell migration	2	15.4	*RET, ZEB2*	73.12	2.44E-02	0.994
GOTERM_BP_FAT	GO:0006112~energy reserve metabolic process	2	15.4	*PHKB, AGL*	62.92	2.83E-02	0.997
GOTERM_BP_FAT	GO:0001838~embryonic epithelial tube formation	2	15.4	*RET, ZEB2*	62.92	2.83E-02	0.997
GOTERM_BP_FAT	GO:0035148~tube lumen formation	2	15.4	*RET, ZEB2*	61.49	2.89E-02	0.998
GOTERM_BP_FAT	GO:0048762~mesenchymal cell differentiation	2	15.4	*RET, ZEB2*	53.05	3.34E-02	0.999
GOTERM_BP_FAT	GO:0014031~mesenchymal cell development	2	15.4	*RET, ZEB2*	53.05	3.34E-02	0.999
GOTERM_BP_FAT	GO:0060485~mesenchyme development	2	15.4	*RET, ZEB2*	52.03	3.41E-02	0.999
GOTERM_BP_FAT	GO:0044264~cellular polysaccharide metabolic process	2	15.4	*PHKB, AGL*	51.05	3.47E-02	0.999
GOTERM_BP_FAT	GO:0016331~morphogenesis of embryonic epithelium	2	15.4	*RET, ZEB2*	46.65	3.79E-02	1.000
GOTERM_BP_FAT	GO:0060562~epithelial tube morphogenesis	2	15.4	*RET, ZEB2*	40.38	4.37E-02	1.000

### Temporal and spatial expression of candidate genes in colon

**Figure 5:**
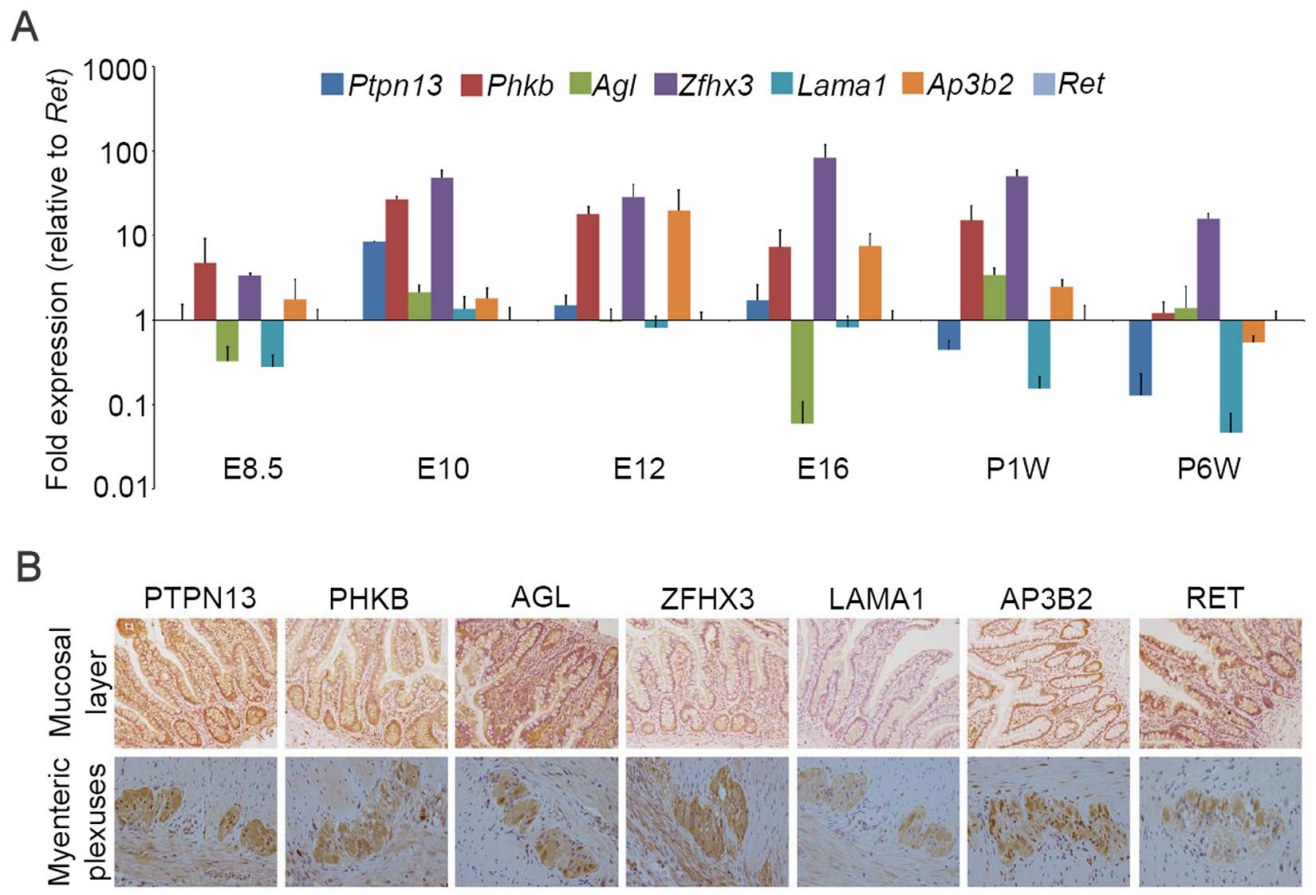
Temporal and spatial expression of candidate genes in the colon. (**A**) Quantitative gene expression of *Ptpn13*, *Phkb*, *Agl*, *Zfhx3*, *Lama1*, *Ap3b2*, and *Ret* in mouse colon tissue during embryonic development. We analyzed gene expression using real-time PCR in fetal (E8.5, E10, E12, and E16) and postnatal (1 and 6 weeks) mice. Replicate experiments were normalized to *Gapdh* as a control. *Ret* was subsequently assigned a value of one for comparison of relative expression levels across the 6 time points. Each gene has been color coded identically across all time points (y axis in log_10_ scale). (**B**) Immunohistochemical staining of colon tissue from two human controls revealed intense expression of PTPN13, PHKB, AGL, ZFHX3, AP3B2, and RET, but not LAMA1 in both the mucosal layer and myenteric plexuses.

To test for possible roles of the candidate genes in ENS development and HSCR pathogenicity, we explored the protein expression and distribution of all 13 LGD_strict_ variant-related genes and *ZFHX3* in normal human colon tissue. Immunohistochemistry was available for 12 genes from the Human Protein Atlas Database (http://www.proteinatlas.org/) and this revealed moderate to strong staining for the proteins (Supp. Fig. S4). Then, 6 genes (*PTPN13*, *PHKB*, *AGL*, *ZFHX3*, *LAMA1*, and *AP3B2*) were prioritized for further validation and their expression in both mouse and human colon was assessed relative to the positive ENS developmental marker *RET*. These genes were selected on the basis of burden test results, functional annotation, novelty, high number of affected cases (recurrent affected patients) in the Chinese population, phenotype severity of the mutation carriers, results from other model organisms, and analysis of the literature indicating an association with neural function and/or embryonic development. We analyzed gene expression using real-time PCR in fetal (E8.5, E10, E12 and E16) and postnatal (1 and 6 weeks) mouse colon tissues, in comparison to *Ret* expression and after normalization to *Gapdh*. More importantly, signals for all 6 genes were present in gut tissues, the strongest signal is from *Zfhx3* and the weakest from *Lama1*. Both *Zfhx3* and *Phkb* showed remarkably high expression levels as early as E8.5 and they steadily increased with development. In contrast, *Ptpn13* and *Agl* only displayed transient expression in the gut at E10 and 1 week postnatal, while the neuron-specific adaptor protein-3 complex encoding gene *Ap3b2* rose dramatically at E12 and then gradually fell to low levels until postnatal week 6 (Fig. 5A). In addition, immunohistochemical staining of colon tissue from the two human controls revealed intense expression for all proteins except LAMA1 in both the mucosal layer and myenteric plexuses (Fig. 5B).

Finally, to investigate whether the candidate variants could affect gene expression, we assessed the expression levels of the proteins PTPN13, PHKB, AGL, ZFHX3, LAMA1, AP3B2, and RET in colon tissue from 16 patients who had been identified as carrying LGD mutations. By comparing with the ganglionic segment, we found that PHKB was downregulated in HSCR stenotic tissues while the others showed no significant differences (data not shown). These results indicated that variants of PHKB might cause different expression of the protein in colon tissue.

## Discussion

HSCR is the most common disorder of the ENS with an incidence of approximately 1/5,000 live births in newborns of European ancestry, and it is twice as common in Asians. Motility disturbances in the distal colon usually lead to neonatal functional intestinal obstruction that needs surgical treatment. Familial occurrence, male predominance, high sibling recurrence risk, and the pattern of associated malformations strongly imply a genetic etiology^2,17^. However, non-mendelian inheritance in human and mouse model studies suggest the presence of significant genetic heterogeneity and multifactorial causes of the disease^18–20^. Mutations in >15 genes are known to be associated with HSCR. Among these, inactivating germline mutations in the *RET* proto-oncogene play the major role in pathogenesis. Currently-available genetic testing is often insufficient as it mainly focuses on *RET* and several other well-known genes that explain only 15–20% of all cases. A high-throughput and cost-effective method is, thus, needed to identify novel, potentially pathogenic variants in additional genes. In this study, we have analyzed a panel of 172 HSCR candidate genes by targeted next-generation sequencing. We have obtained a satisfying sequence coverage with ~92% of all coding bases covered by >10 reads (except for HSCR0042, of whom we may have lost power to detect causal variants), although the 20x performance still needs significant improvement for reliable mutation screening in the future. In a cohort of 83 Chinese HSCR patients, we identified 19 possibly pathogenic variants in these 172 genes. As expected, the diagnostic yield was higher among the long-segment and TCA patients (21.6%) than that of the short-segment patients (15.6%).

To maximize the possibility of detecting mutations and/or genes that convincingly contribute to HSCR, we used a two-step variant filtering and prioritization approach, considering both the effect and literature-based evidence of a variant. Two categories of variants were defined with different criteria: LGD_strict_ and LGD_broad_. We first analyzed the distribution of the LGD_strict_ variants across different HSCR categories. As expected, a higher frequency of these variants was found among patients with the most severe forms of the disease (11/16 HSCR patients had L-HSCR + TCA, compared with 5/16 patients with S-HSCR), which is in accord with a previous report^2^. However, the highest frequency of LGD_strict_ variants was found among males, contrary to the anticipation that the affected individuals from the less frequently affected sex (females for HSCR) should have a higher mean liability and thus should carry more susceptibility alleles. We speculate that these results might have been affected by two important confounders: the limited sample size, and the gender composition bias of the patients recruited (male to female 1.59).

Altogether, 19 LGD_strict_ variants were detected in 13 genes affecting 16 patients, among which *RET* was the only recurrent disrupting gene affecting 7 unrelated isolated patients. Parental DNA were available from 5 families and all 5 mutations (p.E252X, p.Y263X, p.R770X, p.Q860X, and c.2802-2 A > G) were demonstrated to be *de novo*. In addition, the nonsense mutation p.Q860X co-segregated with the phenotype where an unaffected younger brother of HSCR0117 was determined to have the wild-type. All these genetic findings reinforce the notion that *RET* is a single, major, necessary causal gene of HSCR. Conversely, pathogenic roles for variants in other genes were uncertain because there was either no phenotype co-segregation, or the collective effect of coding mutations in each individual gene did not survive the burden test, or the existence of numerous singletons made recurrence of a certain variant extremely unlikely.

Fortunately, the analysis of gene sets and gene networks provided an effective alternative solution, which could aggregate a sufficient number of rare variants for an enrichment analysis to reach statistical significance. GeneMANIA revealed an interconnected network of 11 out of 13 proteins through either co-expression, co-localization, shared protein domains, or predicted interactions, strengthening the possibility of epistatic, synergistic, and/or combining the functions of multiple genes contributing to HSCR susceptibility. In addition, functional annotation and cluster analysis showed that HSCR genes tended to enrich in the regulation of ENS development (*RET* and *TLX2*), glucose, hexose, monosaccharide metabolism (*PHKB*, *ENO3*, and *AGL*), tissue morphogenesis, neural crest cell differentiation, and development (*RET* and *ZEB2*). It is well known that metabolic disturbance during or before pregnancy is associated with several birth defects^21,22^, but the discovery of enriched genes associated with glucose metabolism in HSCR, especially in the patients themselves, was quite surprising. Recently, many studies have investigated the roles of hypothyroidism, vitamin A deficiency, and maternal medicine usage during pregnancy in HSCR^23–26^, but none of these associations have been confirmed and the importance of environmental factors remains unclear. We noted that a population-based case-control study in Sweden revealed that maternal obesity increases the risk for a child to have HSCR^27^. We speculate that an unbalanced or disrupted metabolism during embryonic development, either through maternal distress or genetic variation in the offspring itself, might increase the risk of developing HSCR. Anyhow, with the availability of larger databases and/or functional studies in the future, we expect that some of these variants will be revealed as causative defects. Bearing in mind that our cohort did not represent a perfect sample of HSCR patients, the gene-set enrichment analysis (GSEA) results should be carefully interpreted before anyone can draw conclusions on the collective contribution of 172 candidate genes in Chinese HSCR patients, and on the prevalence of mutations in individual HSCR genes. Except *RET*, a transcription factor with vital roles in regulating myogenic and neuronal differentiation, seems to be a plausible candidate: both the temporal and spatial expression patterns of *ZFHX3* in mouse and human colon suggest its possible role in the pathogenesis of HSCR. In any case, follow-up functional experiments are required to determine its *in vitro* and *in vivo* effects on the phenotype.

In conclusion, targeted next generation sequencing-based analysis is a powerful tool to detect disease-causing pathological variants in HSCR patients. The results of this study broaden the mutational spectrum of HSCR candidate genes, and they provide insight into the relative contributions of individual genes to this highly heterogeneous disorder. The application of this approach to a larger patient cohort will provide the basis for a comprehensive and detailed genetic landscape of HSCR, especially in China.

## Electronic supplementary material

Supplementary Information
