# Supplementary material for: Sporadic Hirschsprung Disease: Mutational Spectrum and Novel Candidate Genes Revealed by Next-generation Sequencing
Source: Sci Rep. 2017 Nov 1;7:14796. doi: 10.1038/s41598-017-14835-6 (PMC5666020; doi:10.1038/s41598-017-14835-6)
Supplement: Supplementary file 1 — Supplementary Information [file 41598_2017_14835_MOESM1_ESM.pdf]

Sporadic Hirschsprung Disease: Mutational Spectrum and Novel Candidate Genes Revealed by Next-generation Sequencing

Zhen Zhang[1], Qi Li[1], Mei Diao[1], Na Liu[2], Wei Cheng[3], Ping Xiao[4], Jizhen Zou[4], Lin Su[5], Kaihui Yu[6], Jian Wu[2], Long Li[1], Qian Jiang[7*]

[1]*Department of General Surgery, Capital Institute of Pediatrics, Beijing, China*

[2]*MyGenostics Inc. Beijing, China*

[3]*Department of Surgery, Beijing United Family Hospital, Beijing, China; Department of Paediatrics and Surgery, Faculty of Medicine, Nursing and Health Sciences, Monash University, Victoria, Australia*

[4]*Department of Pathology, Capital Institute of Pediatrics Affiliated Children's Hospital, Beijing, China*

[5]*Reproductive Medicine Center, Clinical College of PLA Affiliated Anhui Medical University, Hefei, China*

[6]*Department of Pathophysiology, School of Preclinical Sciences, Guangxi Medical University, Nanning, China*

[7]*Department of Medical Genetics, Beijing Municipal Key Laboratory of Child Development and Nutriomics, Capital Institute of Pediatrics, Beijing, China*

Supplementary Materials

Supplementary Table Legends:

Table S1: List of the 172 selected genes in targeted next-generation sequencing among Chinese HSCR patients.

Table S2: Quality of the targeted next-generation sequencing data.

Table S3: Comparison of the frequency, type and novelty of likely gene-disrupting variants in 13 genes in the HSCR cases and controls.

Table S4: Results of sequence kernel association test on LGD_{broad} variants implicating genes with a FDR <0.05.

Supplementary Figure Legends:

Fig. S1 Variant composition and distribution across 8 categories (frameshift, in-frame, intronic, non-synonymous, splicing, stop-gain, synonymous, and UTR) before (A) and after (B) the two-step variant filtering process.

Fig. S2 IGV screen-shot of the targeted next-generation sequencing of *FARP1* (A) and the Sanger-sequence chromatogram (B) of HSCR0048.

Fig. S3 Sample and LGD_{broad} variant spectrum. x axis, 83 sample IDs; y axis, gene symbols for LGD_{broad} variants. LGD_{broad} variants are composed of: frameshift indels (red), in-frame indels (blue), non-synonymous variants predicted to be damaging by at least three bioinformatics tools (green), splice region (±5) variants (purple), and stop-gain variants (orange).

Fig. S4 Expression and spatial distribution of 12 proteins in normal human colon tissue. Images were downloaded from The Human Protein Atlas database (http://www.proteinatlas.org/).

Supp. Table S1. List of the 172 selected genes in targeted next-generation sequencing among Chinese HSCR patients

No.	*Official symbol*	*Chromosome location*	*Entrez gene ID*	*Protein size (No. of amino-acids)*	*Selection criteria*	*References*
1	*RET*	10q11.21	5979	1114	Known HSCR genes (evidence from human study)	1-5
2	*EDNRB*	13q22.3	1910	442		
3	*SEMA3D*	7q21.11	223117	777		
4	*SEMA3C*	7q21.11	10512	751		
5	*NRG1*	8p12	3084	640		
6	*PHOX2B*	4p13	8929	314		
7	*SOX10*	22q13.1	6663	466		
8	*ECE1*	1p36.12	1889	770		
9	*EDN3*	20q13.32	1908	238		
10	*GDNF*	5p13.2	2668	211		
11	*KIF1BP (KIAA1279)*	10q22.1	26128	621		
12	*L1CAM*	Xq28	3897	1257		
13	*NRTN*	19p13.3	4902	197		
14	*TCF4*	18q21.2	6925	667		
15	*ZEB2*	2q22.3	9839	1214		
16	*NAV2*	11p15.1	89797	2488	Known HSCR candidate genes (evidence from CNV or gene expression study)	6-8
17	*WWOX*	16q23.1	51741	414		
18	*KIAA0368*	9q31.3	23392	1845		
19	*PLEKHA1*	10q26.13	59338	404		
20	*MAPK10*	4q21.3	5602	464		
21	*LARGE 1 (LARGE)*	22q12.3	9215	756		
22	*SYN3*	22q12.3	8224	580		
23	*GRIN2B*	12p13.1	2904	1484		
24	*LRRTM4*	2p12	80059	590		
25	*SOX2*	3q26.33	6657	317		
26	*NCRNA00158 (LINC00158)*	21q21.3	54072	81		

27	*DLX2*	2q31.1	1746	328	Known HSCR candidate genes (evidence from mouse study)	9-13
28	*GFRA1*	10q25.3	2674	465		
29	*ARAF*	Xp11.23	369	606		
30	*GRB10*	7p12.1	2887	594		
31	*HOXA2*	7p15.2	3199	376		
32	*PHACTR4*	1p35.3	65979	702		
33	*TLX2*	2p13.1	3196	284		
34	*ZIC2*	13q32.3	7546	532		
35	*GPR98 (ADGRV1)*	5q14.3	84059	6306	New HSCR candidate genes (evidence from the WES study &ENS expression)	
36	*UBR4*	1p36.13	23352	5183		
37	*FAT1*	4q35.2	2195	4588		
38	*SACS*	13q12.12	26278	4579		
39	*DST*	6p12.1	667	7570		
40	*POLE*	12q24.33	5426	2286		
41	*IFIH1*	2q24.2	64135	1025		
42	*ODZ3 (TENM3)*	4q35.1	55714	2699		
43	*BIRC6*	2p22.3	57448	4857		
44	*ZFHX3*	16q22.3	463	3703		
45	*AGL*	1p21.2	178	1532		
46	*COL11A1*	1p21.1	1301	1806		
47	*MICAL2*	11p15.3	9645	1124		
48	*ATM*	11q22.3	472	3056		
49	*TBC1D9B*	5q35.3	23061	1250		
50	*LAMA1*	18p11.3	284217	3075		
51	*APC*	5q22.2	324	2843		
52	*ANK3*	10q21.2	288	4377		
53	*SORL1*	11q24.1	6653	2214		
54	*PTPN13*	4q21.3	5783	2485		
55	*SPTAN1*	9q34.11	6709	2472		
56	*TULP4*	6q25.3	56995	1543		

57	*KANK1*	9p24.3	23189	1352
58	*NID2*	14q22.1	22795	1375
59	*TRRAP*	7q22.1	8295	3859
60	*DMD*	Xp21.2	1756	3685
61	*DMXL2*	15q21.2	23312	3036
62	*MPDZ*	9p23	8777	2070
63	*AGRN*	1p36.33	375790	2067
64	*CFTR*	7q31.2	1080	1480
65	*MDN1*	6q15	23195	5596
66	*KALRN*	3q21.2	8997	2985
67	*HERC1*	15q22.31	8925	4861
68	*FLNB*	3p14.3	2317	2602
69	*ACSS2*	20q11.22	55902	701
70	*BAZ2B*	2q24.2	29994	2168
71	*BCMO1*	16q23.2	53630	547
72	*CHD1*	5q15	1105	1710
73	*COL14A1*	8q24.12	7373	1796
74	*DSP*	6p24.3	1832	2871
75	*EML4*	2p21	27436	981
76	*FRMD4B*	3p14.1	23150	1034
77	*LRP6*	12p13.2	4040	1613
78	*MAGI3*	1p13.2	260425	1506
79	*PLEKHH1*	14q24.1	57475	1364
80	*RANBP17*	5q35.1	64901	1088
81	*TSR1*	17p13.3	55720	804
82	*ARVCF*	22q11.21	421	962
83	*SULF1*	8q13.3	23213	871
84	*NUP155*	5p13.2	9631	1391
85	*ADAMTS15*	11q24.3	170689	950
86	*KIAA1109*	4q27	84162	5005

87	*TRAP1*	16p13.3	10131	704
88	*FBN2*	5q23.3	2201	2912
89	*UTP20*	12q23.2	27340	2785
90	*VPS13C*	15q22.2	54832	3753
91	*MYOF*	10q23.33	26509	2061
92	*MACF1*	1p34.3	23499	7388
93	*AARS*	16q22.1	16	968
94	*AP3B2*	15q25.2	8120	1082
95	*APPL2*	12q23.3	55198	664
96	*ASTN1*	1q25.2	460	1302
97	*ATG2B*	14q32.2	55102	2078
98	*COL6A2*	21q22.3	1292	1019
99	*CPNE1*	20q11.22	8904	537
100	*CREBBP*	16p13.3	1387	2442
101	*ENO3*	17p13.2	2027	434
102	*EPB41L3*	18p11.31	23136	1087
103	*EPHA7*	6q16.1	2045	998
104	*ERCC3*	2q14.3	2071	782
105	*GSN*	9q33.2	2934	782
106	*PATJ (INADL)*	1p31.3	10207	1801
107	*MMP1*	11q22.2	4312	469
108	*MYH9*	22q12.3	4627	1960
109	*NEDD9*	6p24.2	4739	834
110	*NUP188*	9q34.11	23511	1749
111	*PKD2*	4q22.1	5311	968
112	*RERE*	1p36.23	473	1566
113	*SH3PXD2A*	10q24.33	9644	1133
114	*SRRM1*	1p36.11	10250	904
115	*TANC1*	2q24.2	85461	1861
116	*TPR*	1q31.1	7175	2363

117	*TRIO*	5p15.2	7204	3097		
118	*VPS16*	20p13	64601	839		
119	*XYLT1*	16p12.3	64131	959		
120	*CAMTA1*	1p36.31	23261	1673		
121	*COL6A3*	2q37.3	1293	3177		
122	*FARP1*	13q32.2	10160	1045		
123	*COLEC12*	18p11.32	81035	742		
124	*HECTD1*	14q12	25831	2610		
125	*FRYL*	4p11	285527	3013		
126	*CAPN2*	1q41	824	700		
127	*PTCH1*	9q22.32	5727	1447		
128	*DYNC1H1*	14q32.31	1778	4646		
129	*PYGB*	20p11.21	5834	843		
130	*IQGAP2*	5q13.3	10788	1575		
131	*LRIG1*	3p14.1	26018	1093		
132	*KIAA1217*	10p12.2	56243	1943		
133	*EHBP1*	2p15	23301	1231		
134	*USP45*	6q16.2	85015	814		
135	*ERCC4*	16p13.12	2072	916		
136	*SVIL*	10p11.23	6840	2214		
137	*CELSR3*	3p21.31	1951	3312		
138	*PYGL*	14q22.1	5836	847		
139	*MYOM1*	18p11.31	8736	1685		
140	*EXO1*	1q43	9156	846		
141	*PHKB*	16q12.1	5257	1093		
142	*UGGT2*	13q32.1	55757	1516		
143	*PAX3*	2q35	5077	479	New HSCR candidate genes (evidence from the ENS development)	14-17
144	*FZD3*	8p21	7976	666		
145	*VANGL1*	1p13.1	81839	524		
146	*VANGL2*	1q22-q23	57216	521		

147	*PRICKLE1*	12q12	144165	831	
148	*PRICKLE2*	3p14.1	166336	844	
149	*DVL1*	1p36	1855	695	
150	*DVL2*	17p13.1	1856	736	
151	*SHH*	7q36	6469	462	
152	*DLL3*	19q13	10683	618	
153	*GLI1*	12q13.2-q13.3	2735	1106	
154	*NOTCH1*	9q34.3	4851	2555	
155	*NOTCH2*	1p13-p11	4853	2471	
156	*DLL1*	6q27	28514	723	
157	*HES1*	3q28-q29	3280	280	
158	*HOXB5*	17q21.3	3215	269	
159	*TCOF1*	5q32	6949	1488	
160	*HMCN1*	1q31.1	83872	5635	New HSCR candidate genes (evidence from the WES study)
161	*DNAJC13*	3q22.1	23317	2243	
162	*LRP1B*	2q22.1	53353	4599	
163	*RNF123*	3p21.31	63891	1314	
164	*GAPVD1*	9q33.3	26130	1478	
165	*INVS*	9q31.1	27130	1065	
166	*USP13*	3q26.33	8975	863	
167	*ABCA1*	9q31.1	19	2261	
168	*ADAMTS17*	15q26.3	170691	1095	
169	*CFAP206 (C6orf165)*	6q15	154313	622	
170	*HHIPL2*	1q41	79802	724	
171	*MERTK*	2q13	10461	999	
172	*UBA6*	4q13.2	55236	1052	

Supp. Table S2. Quality of the targeted NGS data

Item	*Clean data (Mb)*	*Aligned (%)*	*Effective sequence on target (Mb)*	*Fraction of effective bases on target (%)*	*Average sequencing depth on target*	*Fraction of target covered at least 10 × (%)*	*Fraction of target covered at least 20 × (%)*
Average	582.5	99.33	276.2	51.40	266.8	92.30	85.20
Maximum	1693.7	99.88	633.9	64.40	614.9	99.50	98.90
Minimum	153.3	97.74	76.9	23.50	74.2	74.90	61.50
Medium	557.1	99.38	273.7	53.10	264.2	92.30	84.80

Supp. Table S3. Comparison of the frequency, type and novelty of likely gene-disrupting variants in 13 genes in the HSCR cases and controls

Gene symbol	***HSCR patients (N=83)***			***Controls (N=316)***		
	Number of LGD$_{strict}$ variants	***Type***	***Novel or reported***	***Number of LGD$_{strict}$ variants***	***Type***	***Novel or reported***
ENO3	1	Stop-gain	rs550460218	0	NA	NA
PTPN13	1	Stop-gain	Novel	0	NA	NA
RET	7	5 Stop-gain<br>1 Frameshift<br>1 Splicing	6 Novel<br>1 Reported (rs775711017)	0	NA	NA
SACS	1	Frameshift	rs761184491	1	Frameshift	rs761200300
PLEKHH1	1	Stop-gain	rs111462449	0	NA	NA
TRAP1	1	Frameshift	Novel	1	Stop-gain	rs371020906
EXO1	1	Stop-gain	Novel	0	NA	NA
ZEB2	1	Stop-gain	Reported (Pathogenic)	0	NA	NA
AGL	1	Stop-gain	rs781580050 (Pathogenic)	1	Frameshift	rs760589837
SEMA3D	1	Stop-gain	Novel	0	NA	NA
PHKB	1	Stop-gain	Novel	0	NA	NA
HHIPL2	1	Frameshift	rs748262144	2	Splicing	rs199911924<br>rs577700102
TLX2	1	Frameshift	Novel	0	NA	NA

NA: not available.

Supp. Table S4. Results of sequence kernel association test on $\mathrm{LGD}_{\mathrm{broad}}$ variants implicating genes with a FDR <0.05

Genes	*P* value	FDR
ZFHX3	2.54E-12	3.32E-10
RET	5.20E-11	3.41E-09
NRG1	4.17E-07	1.82E-05
CFTR	6.84E-07	2.24E-05
AGL	4.11E-05	0.001077
IQGAP2	9.69E-05	0.002116
LAMA1	0.000332	0.005641
NID2	0.000377	0.005641
RERE	0.000388	0.005641
IFIH1	0.000714	0.009359
MYOF	0.000993	0.011829
APC	0.001155	0.012067
ZEB2	0.001208	0.012067
PTPN13	0.00129	0.012067
DMD	0.001659	0.014491
TRAP1	0.001857	0.015202
CELSR3	0.002226	0.01715
ADAMTS17	0.004172	0.01793
PHACTR4	0.004194	0.01793
KANK1	0.004208	0.01793
NUP155	0.004229	0.01793
EML4	0.004252	0.01793
FARP1	0.004258	0.01793
CREBBP	0.004259	0.01793
NOTCH2	0.004268	0.01793
CFAP206	0.004297	0.01793
FBN2	0.0043	0.01793
SH3PXD2A	0.004316	0.01793
ABCA1	0.004322	0.01793
AP3B2	0.004413	0.01793
HMCN1	0.004447	0.01793
ARVCF	0.004579	0.01793
TSR1	0.004589	0.01793
FLNB	0.004654	0.01793
PATJ	0.005278	0.019755
COL11A1	0.006813	0.024792
SORL1	0.008282	0.029113
NOTCH1	0.008445	0.029113
AGRN	0.011116	0.029871
EXO1	0.013108	0.029871

UBR4	0.013383	0.029871
ADGRV1	0.013506	0.029871
PYGB	0.013561	0.029871
MICAL2	0.013584	0.029871
TENM3	0.013672	0.029871
BIRC6	0.013738	0.029871
ECE1	0.013805	0.029871
PRICKLE1	0.013828	0.029871
ENO3	0.013861	0.029871
PLEKHH1	0.013873	0.029871
HERC1	0.013894	0.029871
APPL2	0.01395	0.029871
SACS	0.013963	0.029871
SRRM1	0.013995	0.029871
DMXL2	0.014051	0.029871
PTCH1	0.014063	0.029871
MYH9	0.014085	0.029871
GRIN2B	0.014085	0.029871
USP45	0.014141	0.029871
SVIL	0.014164	0.029871
PHKB	0.014286	0.029871
ATM	0.014355	0.029871
XYLT1	0.014365	0.029871
EHBP1	0.014714	0.030118
KIAA1109	0.021628	0.04359
MACF1	0.024251	0.048052
VPS13C	0.024576	0.048052

Supplementary Fig. S1

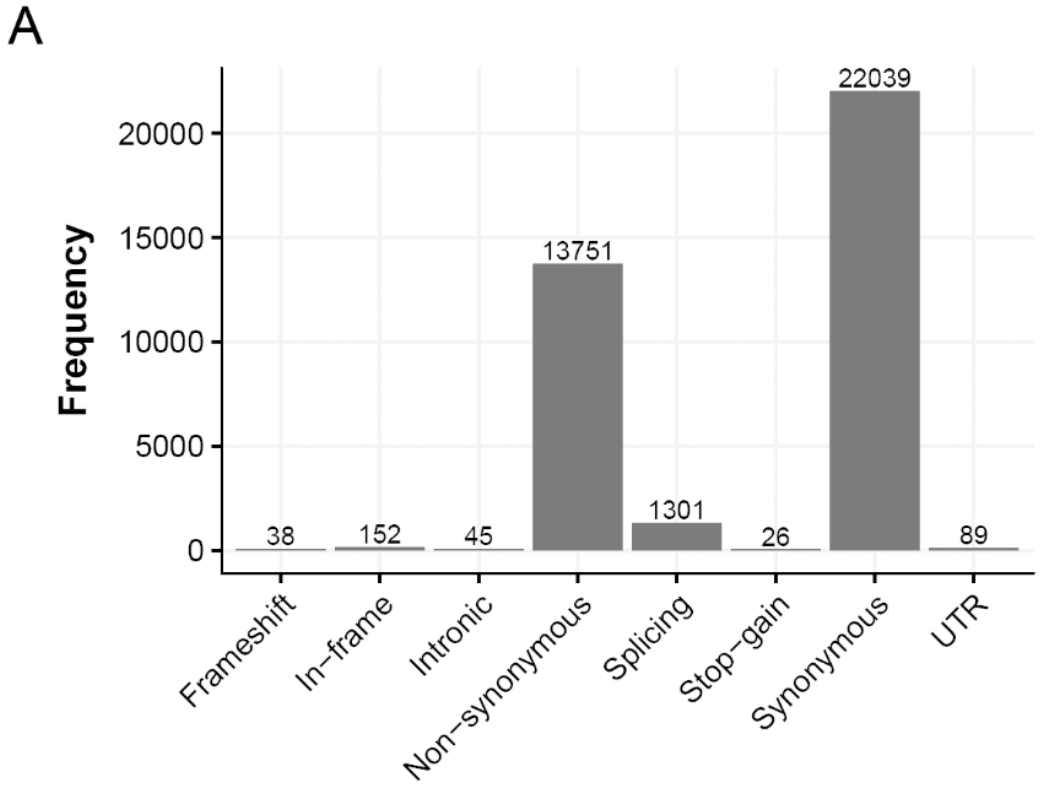

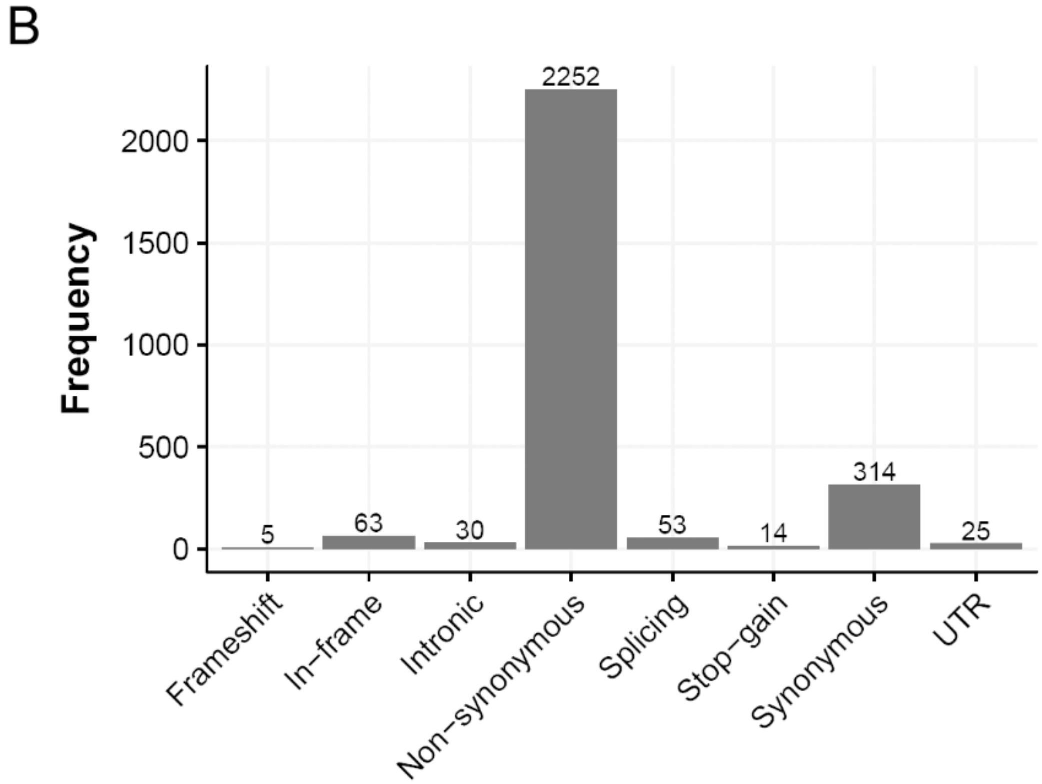

Supplementary Fig. S2

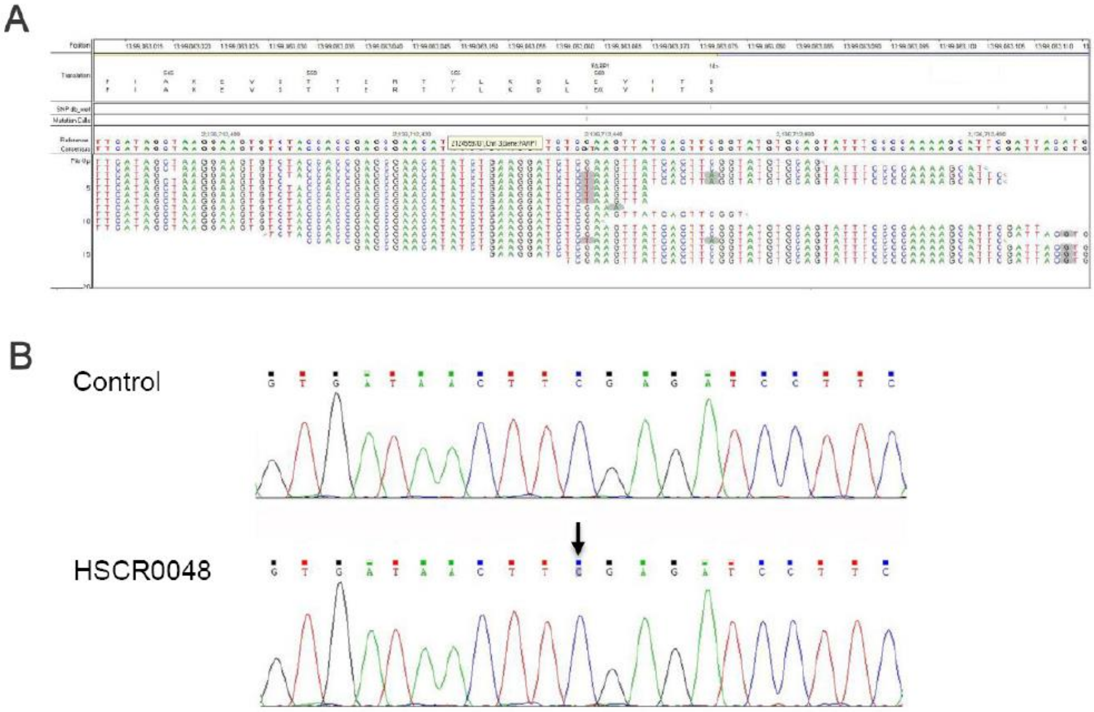

Supplementary Fig. S3

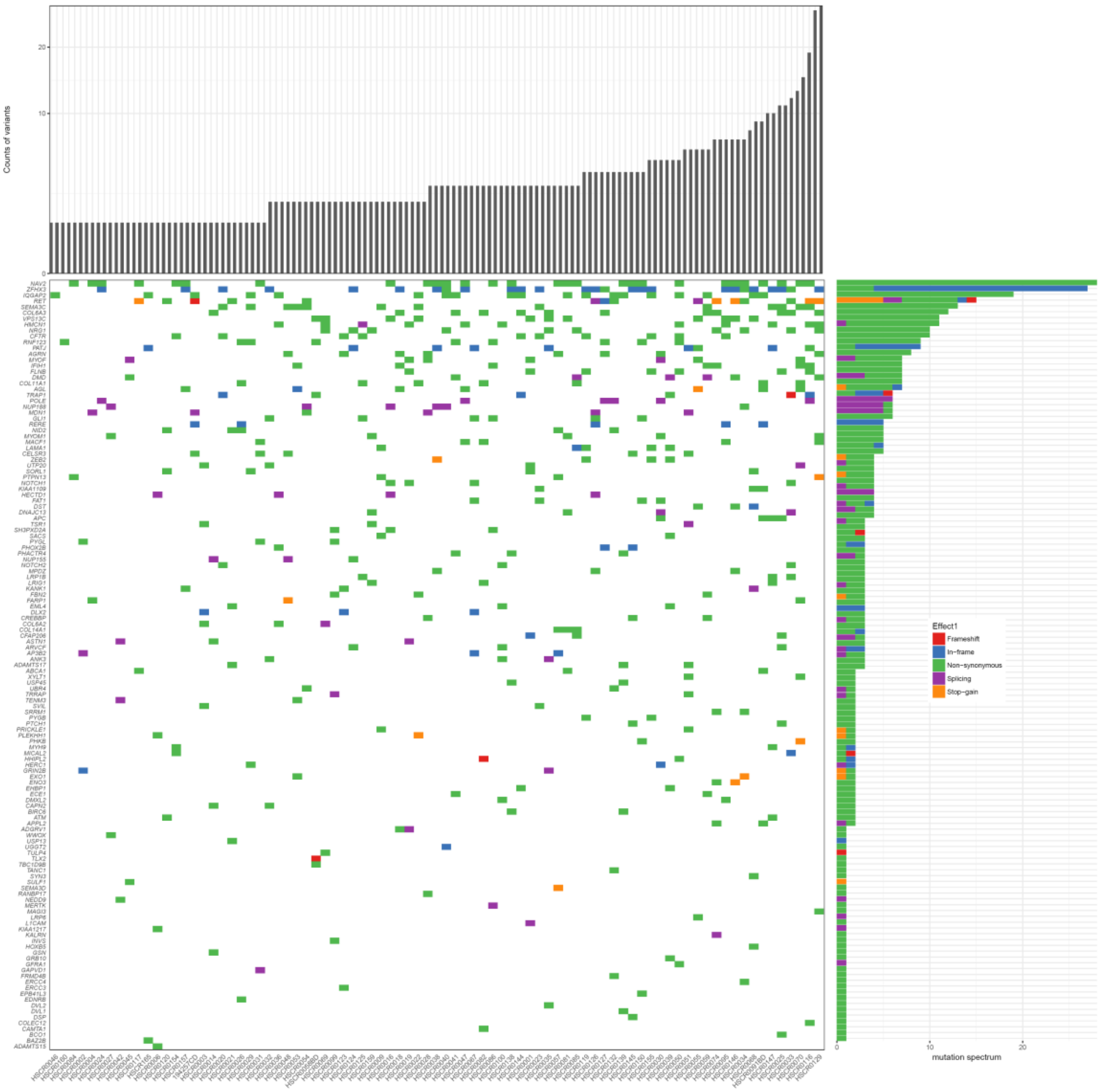

Supplementary Fig. S4

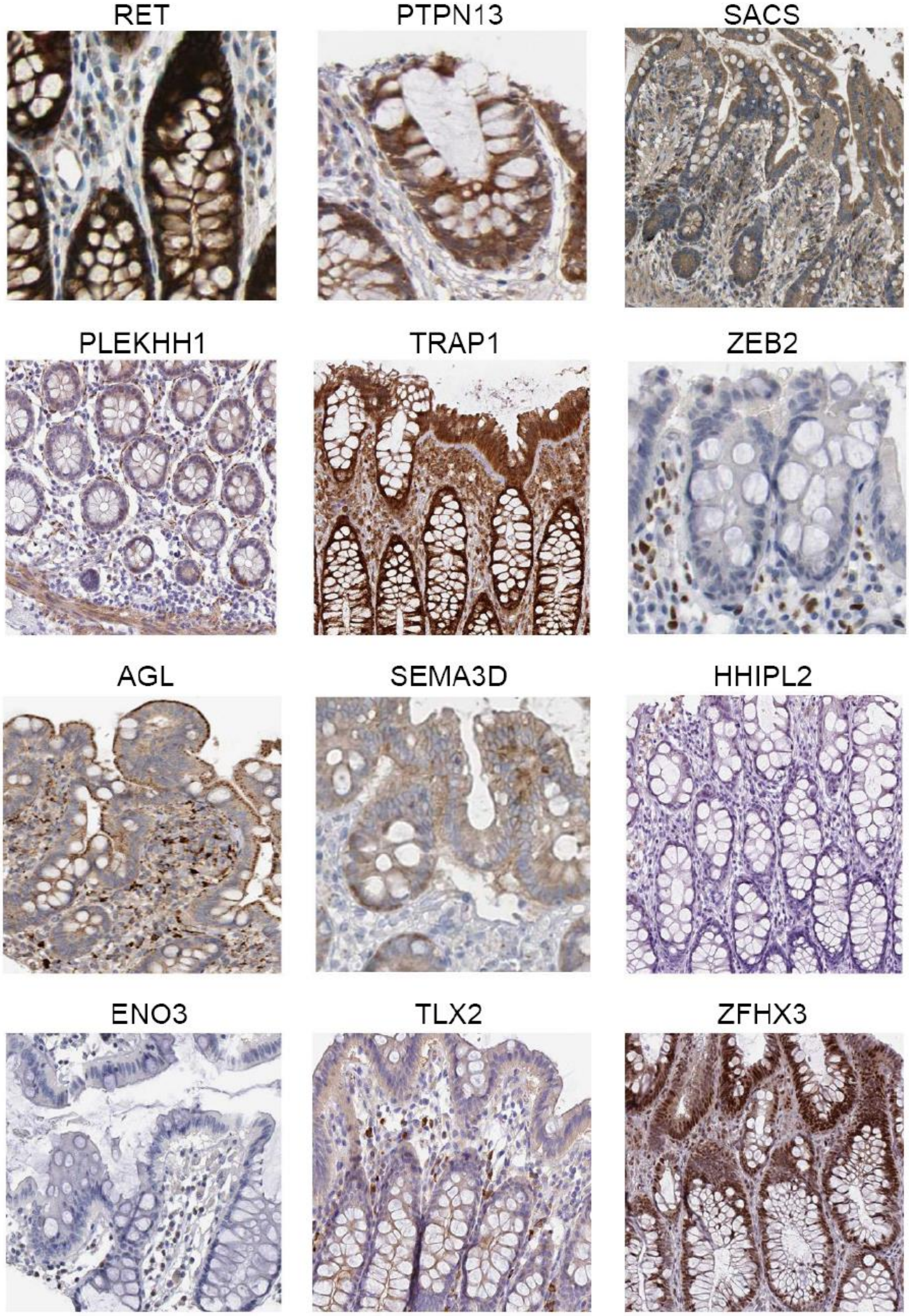

References

1. Chatterjee, S. *et al.* Enhancer variants synergistically drive dysfunction of a gene regulatory network in Hirschsprung disease. *Cell.* **167**, 355-368 (2016).
2. Jiang, Q. *et al.* Functional loss of semaphorin 3C and/or semaphorin 3D and their epistatic interaction with ret are critical to Hirschsprung disease liability. *Am. J. Hum. Genet.* **96**, 581-596 (2015).
3. Borrego, S., Ruiz-Ferrer, M., Fernández, R. M., & Antiñolo, G. Hirschsprung's disease as a model of complex genetic etiology. *Histol. Histopathol.* **28**, 1117-1136 (2013).
4. Garcia-Barcelo, M. M. *et al.* Genome-wide association study identifies NRG1 as a susceptibility locus for Hirschsprung's disease. *Proc. Natl. Acad. Sci. USA.* **106**, 2694-2699 (2009).
5. Brooks, A.S. *et al.* Homozygous nonsense mutations in KIAA1279 are associated with malformations of the central and enteric nervous systems. *Am. J. Hum. Genet.* **77**, 120-126 (2005).
6. Nunez, M. I., Ludes-Meyers, J., & Aldaz, C. M. WWOX protein expression in normal human tissues. *J. Mol. Histol.* **37**, 115-125 (2006).
7. Sherwood, R. I., Chen, T. Y., & Melton, D. A. Transcriptional dynamics of endodermal organ formation. *Dev. Dyn.* **238**, 29-42 (2009).
8. Jiang, Q., Ho, Y. Y., Hao, L., Nichols, Berrios, C., & Chakravarti, A. Copy number variants in candidate genes are genetic modifiers of Hirschsprung disease. *PLoS. One.* **6**, e21219 (2011).
9. Heanue, T.A., & Pachnis, V. Expression profiling the developing mammalian enteric nervous system identifies marker and candidate Hirschsprung disease genes. *Proc. Natl. Acad. Sci. USA.* **103**, 6919-6924 (2006).
10. Pritchard, C. A., Bolin, L., Slattery, R., Murray, R., & McMahon, M. Post-natal lethality and neurological and gastrointestinal defects in mice with targeted disruption of the A-Raf protein kinase gene. *Curr Biol.* **6**, 614-617 (1996).
11. Hatano, M. *et al.* A novel pathogenesis of megacolon in Ncx/Hox11L.1 deficient mice. *J. Clin. Invest.* **100**, 795-801 (1997).
12. Zhang, Y., Kim, T.H., & Niswander, L. Phactr4 regulates directional migration of enteric neural crest through PP1, integrin signaling, and cofilin activity. *Genes. Dev.* 2**6**, 69-81 (2012).
13. Elms, P., Siggers, P., Napper, D., Greenfield, A., & Arkell, R. Zic2 is required for neural crest formation and hindbrain patterning during mouse development. *Dev. Biol.* 2**64**, 391-406 (2003).
14. Ngan, E. S. *et al.* Hedgehog/Notch-induced premature gliogenesis represents a new disease mechanism for Hirschsprung disease in mice and humans. *J. Clin. Invest.* **121**, 3467-3478 (2011).
15. Jia, H., Zhang, K., Chen, Q., Gao, H., & Wang, W. Downregulation of Notch-1/Jagged-2 in human colon tissues from Hirschsprung disease patients. *Int. J. Colorectal. Dis.* 2**7**, 37-41 (2012).
16. Sasselli, V., Boesmans, W., Vanden, Berghe, P., Tissir, F., Goffinet, A. M., & Pachnis, V. Planar cell polarity genes control the connectivity of enteric neurons. *J. Clin.*

Invest. **123**, 1763-1772 (2013).

17. Obermayr, F., Hotta, R., Enomoto, H., & Young, H. M. Development and developmental disorders of the enteric nervous system. *Nat. Rev. Gastroenterol. Hepatol.* **10**, 43-57 (2013).
